# A Review of the Evidence on Attitudes, Perceived Impacts and Motivational Factors for European Member State Collaboration for Pricing and Reimbursement of Medicines: Time for the EEA Member States to Apply Their Experience and Expertise in Evidence-Based Decision Making to Their Current Pharmaceutical Policy Challenges

**DOI:** 10.3389/fphar.2021.666405

**Published:** 2021-11-12

**Authors:** Patricia Vella Bonanno, Vincent Cassar, Brian Godman

**Affiliations:** ^1^ Department of Management, Faculty of Economics, Management and Accountancy, University of Malta, Msida, Malta; ^2^ Strathclyde Institute of Pharmacy and Biomedical Sciences, University of Strathclyde, Glasgow, United Kingdom; ^3^ Division of Public Health Pharmacy and Management, School of Pharmacy, Sefako Makgatho Health Sciences University, Pretoria, South Africa; ^4^ School of Pharmaceutical Sciences, Universiti Sains Malaysia, Penang, Malaysia

**Keywords:** pharmaceutical policy, international collaboration, pricing and reimbursement, medicines, evidence-based management, EEA member states, attitudes, perceived impacts and motivational factors

## Abstract

In 2018/2019 there were a number of initiatives for collaboration between Member States in the European Economic Area (EEA) and the European Commission published a Proposal for a Regulation on Health Technology Assessment. In view of the perceived benefits from collaboration, the experiences and challenges of these collaborative initiatives and the possible implications of the proposed legislation, a study of the evidence on attitudes, perceived impacts and the motivational factors towards European Member State collaboration regarding the pricing and reimbursement of medicines was conducted. This study adopted an evidence–based management approach by Barends and Rousseau. The main findings showed that Member States differed in their motivation for collaboration for different pharmaceutical activities. Member States favoured voluntary co-operation for all activities of pricing and reimbursement except for relative effectiveness assessments where Member State authorities had divergent attitudes and prioritised activities related to the sustainability of their healthcare systems and access to medicines. Contrastingly pharmaceutical companies strongly favoured mandatory cooperation for evaluation. Member States motivation for collaboration was highly dependent on the purpose, political will, implementation climate and cultural factors. Currently, with the experiences of ongoing collaborations, following the progress of the discussion at Council, and with a number of inititatives for new pharmaceutical strategy and policy, it is proposed that Member States use their trust, expertise and knowledge of application of evidence-based decision making for pricing and reimbursement of medicines and apply it to decide the future model for Member State collaboration. The applicability of principles of evidence-based management to pharmaceutical policy can be used as a starting point.

## Introduction

### Member State Collaboration for Pricing and Reimbursement of Medicines

The area of new medicines is very significant for European Member States (MSs). Continued high prices for new medicines particularly for orphan diseases and cancer affect the sustainability of European healthcare systems (Godman et al., 2018; Luzzatto et al., 2018). Access and affordability of new medicines are a challenge for all MSs to different levels, leading to appreciable variation in their use across Europe, which is a concern where equity of care is a strong principle (EURORDIS 2018; Baumgart et al., 2019; Wilking et al., 2019). Access to affordable medicines is one of the objectives of universal health coverage and of the World Health Organisation (WHO) sustainable development goals (Hogan et al., 2018). All governments within the European Union, including high-income countries, are experiencing increasing difficulty to provide sustainable access to medicines (Jorgensen and Kefalas 2017; Shukla et al., 2019). Finances will become even more challenging post COVID-19 pandemic with its consequences (Kluge et al., 2020).

A number of initiatives have been instigated by governments and by public authorities for pricing, reimbursement and funding of new premium priced medicines. These initiatives have included innovative models to improve the managed entry of new medicines, horizon scanning, new ways of financing innovative medicines, strategies to improve prescribing and medicines use, as well as post-launch activities and initiatives for regional collaboration between MSs (World Health Organisation 2015; Eriksson et al., 2017; Godman et al., 2018; Vogler et al., 2018; World Health Organisation 2020; Godman et al., 2021).

MS collaboration in the area of pricing and reimbursement is a paradigm shift in the pharmaceutical framework. A number of MSs consider that by collaborating together they can benefit from increasing access to new medicines. This is achieved by developing a stronger stakeholder position within the pharmaceutical policy framework and through augmented negotiation power, particularly with pharmaceutical companies increasingly entering into managed entry agreements including confidential discounts (Carlson et al., 2017; Ferrario et al., 2017; Dias et al., 2020).

In the last 25 years, there have been a number of initiatives among MSs for collaboration. These were mainly driven by the MSs, were voluntary and were sporadic and lacked coordination between them (World Health Organisation 2015). Council Conclusions of different “Presidencies of the Council of the European Union” supported exclusively MS driven voluntary cooperation on health technology assessment (HTA), advocated for collaboration between groups of MSs and encouraged the sharing of HTA methodologies and assessments of outcomes. They linked voluntary collaboration as a means for improvement of access to medicines (Council of the European Union 2016; Council of the European Union 2017).

The European Commission tried to achieve coordination, and there were several initiatives to this effect. Pricing and reimbursement authorities of most MSs collaborated on a voluntary basis within the “European Network for Health Technology Assessment” (EUnetHTA), a network of governmental organisations, regional agencies and non-profit organisations which collaborate on HTA. EUnetHTA achieved a number of joint outputs particularly tools, guidelines and methodologies for HTA and formed a number of Joint Actions (JA) to support research, communication and work between the collaborators (EUnetHTA 2018). EUnetHTA experienced difficulties including a number of MSs did not use the joint assessments for national decision making, MSs implemented different national HTA models, technical expertise and capacity for HTA differed between MSs, MSs wanted to retain their national method for HTA and the model of EUnetHTA was considered to be economically unsustainable (European Commission 2016). The funding for EUnetHTA is now at an end.

In 2016 the European Commission started studying alternative long-term solutions for MS collaboration for HTA. The Final Report (European Union 2017), henceforth referred to as “the Study,” was commissioned by the European Union (Vella Bonanno et al., 2019). This considered five Policy Options (PO), which were set by the European Commission at three levels of governance: base-line (no EU action); voluntary cooperation without legislation and mandatory cooperation covered by EU legislation.

A “Proposal for a Regulation of the European Parliament and of the Council on health technology assessment and amending Directive 2011/24/EU” (European Commission 2018), henceforth referred to as the “Proposal for a Regulation on HTA,” was published by the EC on 31st of January 2018. The Proposal was amply discussed at Council. MS showed strong divergent positions on the Proposal. By the time of publication of this paper, the outcome of this Proposal was not finalised by Council, but the final product is expected to be significantly different from the original proposal.

In parallel, initiatives for the development of “regional co-operations” within groups of MSs were implemented including the Valletta Declaration, BeNeLuxAIr, FINOSE and Fair Pricing Initiatives. These were voluntary and collaborated on different activities according to priorities. (Espin et al*.* 2016; World Health Organisation, 2020). These collaborations were criticised for making limited progress (EFPIA, 2019).

Different bodies such as the European Commission and the World Health Organisation office for Europe are drafting proposals for pharmaceutical strategies. The European Commission has just launched a document for a “Pharmaceutical Strategy for Europe” (European Commission 2020b) while the World Health Organisation is also working on a proposal for pharmaceutical strategy. Collaboration by Member States and access to medicines seem to be common themes being prioritised in all ongoing proposals.

During the COVID-19 pandemic, MS collaborated in joint procurement for different medical supplies, for vaccines and for remdesivir (European Commission 2020a).

### Scope of the Study

This study aims to collect and present evidence about the specific attitudes, perceived impacts and motivational factors for MS collaboration for pricing and reimbursement of medicines. It focuses on Member States within the European Economic Area (EEA), operating within the framework of EU pharmaceutical policy and legislation.

The first objective was to formulate the theoretical framework to identify the themes for the key topics studied: attitudes, perceived impacts and motivational factors for MS collaboration (*Presented in Theoretical Framework for Collaboration*).

The second objective was to collect and present the evidence about MS collaboration using these themes (Refer to *Method* and *Results*).

This knowledge can support evidence-based decisions about the feasibility and prioritisation of proposed policies related to MS collaboration. It can inform models, decisions and initiatives for MS collaboration in future pharmaceutical strategy for the European Economic Area and also possibly at international level.

## Theoretical Framework for Collaboration

### Regulatory Considerations for Member State Collaboration

The formation and workings of collaborations involve primarily regulatory considerations, which are formalised through legislation or through policies and strategies.

Baldwin et al. (2012) described four main considerations for successful regulation, which the authors of this paper considered to be directly applicable to MS collaboration. The first consideration was that regulators should prioritise public interest, “interest centred approaches.” The second consideration was competition between concerns and interests of different stakeholders involved, which can lead to regulatory failure. Organised stakeholder groups influence regulation to meet their interests. Economically powerful and concentrated interests have the ability to manipulate regulations. Politicians’ behaviour shows that governments change their minds over time (“the time inconsistency problem”). Some regulatory agencies also adopt blame- and risk- avoiding behaviours and focus on achieving popular outcomes rather than those that are significant and often difficult and unpopular. It is important that there is alignment between the organisational self-interest and regulatory alignment. The third consideration was the “ideas-based approaches,” which showed how beliefs, ideas and world views impact regulation. The fourth consideration concerned “institutional theories,” which agree that regulatory developments are driven by institutional structures and arrangements and by social processes. Failure of recognising this will result in inter- and intra-institutional pressures.

Baldwin et al. (2012) stress that it is important that regulatory systems do not “drift” and lose focus and direction. Regulatory authorities can undergo different types of “drifts” including “coalition drifts” (governments changing preferences over time), “agency drift” (agencies not following their statutory objectives) and “industry drifts” (industry not following regulatory requirements) (Baldwin et al., 2012). Information asymmetry may lead to drift.

The regulatory function of organisations is highly linked to the process of decision making of the organisation. The “principle of bounded rationality” by Simon (1990) shows how the decision-making process impacts the decisions made and considers the cognitive limitations of the decision maker. It considers shortcomings in evidence as well as in computational capacity (Simon 1990). As described by Baldwin et al. (2012) bounded rationality “affects individual and organisational decision making. Information is costly and the capacity of any one individual, organisation, or system to process all available information within time and other constraints is inherently limited. As a result, human decision-making is inherently bounded.” It is considered that uncertainty and ambiguity of knowledge can result in limitations of regulation. There needs to be consideration that there are differences in context, legal systems, political systems and constituencies. This limitation in knowledge results in reduced prediction that the regulatory strategies will achieve their intended effect. Baldwin et al. (2012) believed that due to limitations of knowledge, regulatory strategies for change should not rely on “grand schemes” but rather on incremental “trial and error” approaches (Baldwin et al., 2012). Thus based on the recommendations of these authors the introduction of initiatives of MS collaboration should be gradual and not through grand strategies or legislations.

Collaboration would require that the organisation has to learn new norms and new roles and established members within the organisation have to learn new things. Simon (1991) explained that learning within an organisation takes place through the learning of the members of the organisation and by enrolling new participants who have new knowledge (Simon 1991). Simon stressed that internal learning is an important component of organisational learning and that this is a social phenomenon. Simon pictured organisations as “systems of interrelated roles” whereby “a role is a system of prescribed decision premises.” Roles tell the members of the organisation how to reason about the problems and decisions they need to take, where they can find official information and evaluative norms, and what techniques to use to process them. Each of the roles in an organisation presumes the correct enactment of the other roles that surround it and interact with it. Thus the organisation is a “role system” (Simon 1991).

### Hurdles and Faciliatators for Member State Collaboration

It is clear there needs to be strong motivators and benefits for national authorities to agree to collaborate voluntarily, and they need to overcome perceived risks and de-motivators. Vigoda-Gadot and Drory (2006) explain that hurdles for collaboration include the fact that national organisations are structured and oriented to work independently and working together will involve a paradigm shift. Moreover most of these organisations are well-established institutions with a long history and stated power. Organisational politics will be a key issue. Professionals and workers within an organisation are often unwilling to bring to the open the political secrets and networks that support their progression and their personal agendas. Another challenge is that globalisation will make the environment more complex and will introduce a degree of uncertainty (Vigoda-Gadot and Drory, 2006). Simon (1990) stressed that in complicated environments people do not adapt easily or at least to the required level (Simon 1990).

For collaboration to succeed, there needs to be communication and networking over time. People tend to collaborate if they have limitations of resources, and collaboration is enhanced by the perception of increased power and output (Kezar 2006). Mohrman et al. (1995, cited by Kezar 2006, p. 809) claimed that “one of the main reasons collaboration fails is that one cannot impose collaboration within a context designed to support individualistic work.” To make collaboration successful, there needs to be “redesign for collaborative work based both on external challenges and pressure and on the documented benefits of working in this manner” (Kezar 2006). Kezar (2006) stressed the need for development of skills for collaboration and also for the “unlearning of non-collaborative skills.” Redesign of organisations requires the support of management regarding a “*strategy, the tasks of the organisation, organisational structure, general processes, developing rewards to incentivise and introduce accountability, and training and empowerment of people to learn collaboration*” (Kezar 2006). Kezar identified other elements which are necessary to foster collaboration. These include culture, values and relationships; interplay of human dynamics; shared values between the groups or a set of values that draw people together. A “*sense of priority from senior executives*” was considered a critical element for successful collaboration (Kezar 2006).

Koenig-Archibugi (2010) consider that cooperation needs to be implemented within a global environment, which impacts the progress and the shape of any cooperation. The global environment can affect public policies through cooperation whereby countries pledge to abide with certain regulatory obligations agreed between governments. International organisations range from organisations which are totally devoid of autonomous power to the “*rationalist institutionalist approach*,” which explains how “*states succeed in cooperating for mutual advantages despite international anarchy*.” Institutionalists support coordination by “*providing a favourable context for bargaining and, crucially, by presenting focal points to negotiators*.” Joint initiatives which have problems with collaboration must be designed to build trust between countries in order to minimise their motivation to abandon agreements. Once international organisations are created, they set the perception of appropriately normative behaviour among their members and this is likely to direct cooperation among the players. One reason for international delegation among countries is blame-management and as a blame-shifting incentive by governments (Koenig-Archibugi 2010).

### Perceived Impacts (Benefits and Risks) From Collaboration

De Freitas et al. (2018) considered the benefits of collaboration between companies, which include improvements in the supply chain, and can be either primary or secondary. Primary benefits included: better planning, greater production, stronger relationships, diversification of product, shorter cycles and smoother product launches. Secondary benefits included: reduced costs, improved customer service, increased sales, competitiveness, better financial performance and more accommodation of the needs of the customer. However, secondary benefits were only achievable subject to realisation of the primary benefits (De Freitas et al., 2018).

The European Commission measures impacts as part of the process for obtaining feedback about a new proposed legislation in line with the “EU Better Regulation Guidelines.” These include a “toolbox” to have timely information on which the Commission bases its decisions. The Better Regulation methodology aims to increase transparency, the evidence-base and the perspectives of stakeholders for establishing EU policies. The guidelines include measurement of the impact of an initiative or an intervention. The final results of an impact assessment are presented in an “impact assessment report,” which should cover three categories of impact: environmental, social and economic impact. The impacts prioritise the perspectives of small and medium enterprises, competitiveness and the stakeholders that will be affected by the initiative (European Commission 2017). “The Study” (European Union, 2017), considered two types of impacts (benefits and losses) from collaboration between national health authorities for pricing and reimbursement social health impacts and economic impacts. The themes for documenting impacts and the relevant indicators were adapted mainly from the Better Regulation Framework of the European Unioin (European Commission, 2017) and from “the Study” (European Union, 2017) and are presented in Table 1.

**TABLE 1:** Themes and relevant indicators for social health impacts and economic impacts for Member State collaboration.

Themes for social health impacts	Indicators
Employment	
Governance, participation and good administration	Indicators:
	i) Impact of collaboration on involvement of different stakeholders in processes
	ii) the responsibilities of public administrations and other organisations at MS level
	iii) the uptake of joint outputs (e.g., HTA reports, early dialogues, tools)
	iv) resource efficiency of processes
	v) the sustainability of European cooperation (sustainability of processes)
Access to social protection and health systems	Indicator:
	The potential effect of collaboration on the access to treatments that could be considered as “innovative”
Sustainability of health systems	Indicators:
	i) the effect of collaboration on the financing of expensive treatments with little or no added value
	ii) the negotiating power of MSs in setting prices
Public health	Overall public health i) Availability of health technologies on the market
	ii) Access to medicines
**Themes for economic impacts**	**Indicators**
Costs The costs related to the processes	Variability in methods and processes currently employed by national health authorities across the EU; possible duplication of efforts; areas for improvement in consistency and transparency in the criteria used for decision making; what clinical and economic evidence is used in processes
Administrative burden	Administrative burden derived from processes:
	Overall administrative burden; repeated processes/products across European countries; time needed for process; complexity of processes e.g., HTA assessment processes
Competitiveness of EU health technology sector	Competitiveness of SMEs; revenues for industry; predictability of national systems in Europe
Innovation and research	Effect of the intervention on: research climate; innovation in the European market; predictability of the market; reduction in fragmentation
International trade innovation and research	
Functioning of the internal market and competition	Fragmentation of the system in Europe; convergence of methodologies; attractiveness of the European market for industry
Consumers	The availability of medical technologies for patients
Macroeconomic environment	Overall economic growth; labour market

### Motivational Factors (Facilitators and Barriers) for Collaboration

De Freitas et al. (2018) in their systematic review classified different types of barriers and motivators for initiatives for collaboration between companies. Their review explained “motivators” as factors external to the company that contribute to the adoption of the initiative; and “barriers” as all elements that hinder the process of implementing an initiative (De Freitas et al., 2018). Although this systematic review did not study national organisations, the characterisation of the concepts identified were considered relevant for this study. Communication and information technologies were considered as facilitators for companies seeking to collaborate, with human resources also considered as another important factor. Barriers were grouped into cultural, physical and behavioural. Cultural barriers included: deficiency in training to achieve new skills and preparedness, variation in aims and objectives, disassociation, lack of integration of new processes, stringent organisations and resistance, lack of accountability and measure of output, lack of support from senior management, incongruence of function, conflicting organisational culture, lack of comprehensive documentation, lack of joint planning, lack of prioritisation of customer service focus and goals, as well as separated problem solving and decision making. Behavioural barriers to collaboration included: lack of trust, resistance to information sharing, problems in information and communication flow, resistance to change and lack of commitment. Physical barriers included: low investments in IT/IS and telecommunications, insufficient financial resources and lack of other investments.

Kezar (2006) focused on collaboration between educational institutions and identified barriers and facilitators for collaboration which were also considered applicable in this situation. External challenges which acted as motivators for collaboration included: difficult financial times, changing demographics, globalisation, increased complexity and the possibility of combining expertise and enhancing the resources and ability of the institution to meet the needs of the changed environment. Barriers for collaboration within educational institutions included the fact that higher educational institutions often acted as independent entities and adopted highly administrative and hierarchical frameworks (Kezar 2006).

Gorriz-Mifsud et al. (2019) identified a number of challenges for coordination of joint management between forest owners. These included: procedures for decision-making, geographical cohesion, building of trust and legitimacy, internal communication and transparency, trade-offs in efficiency and equity, local idiosyncrasy, dynamics of the management committee, flexibility as compared to risk aversion, legal considerations, long-term vision and joint motivation (Gorriz-Mifsud et al., 2019). These can also be relevant to collaboration between MS entities.

Haines and Donald (2002) listed potential barriers to change in clinical practice settings. Those most relevant included: practice and healthcare environment (e.g., limitation of time, financial resources), educational environment and social environment (e.g., influence of media) (Haines and Donald, 2002).

As motivational factors can be positive or negative, and the same factor can be considered through different perspectives by different stakeholders, “motivational factors” are considered generically in this paper. The framework of the main motivational factors which can act as barriers or facilitators for collaboration are summarised in Table 2.

**TABLE 2:** Motivational factors which act as barriers (negative motivators, challenges) and factors which act as facilitators (positive motivators, drivers) for collaboration between national health authorities for pricing and reimbursement.

**Social** Access to social protection and health systems, sustainable health systems, access to medicines
**Economic** External factors arising from an economic factor or by a market event e.g., more intense competition, globalisation, market reaction, competitive advantage
**Behavioural** Trust, ability or willingness to share information, resistance to change, mutual respect, ability to compromise, communication, personal interests
**Organisational** Internal factors related to the form of organisation: the willingness of the organisation to collaborate; the need for the organisation to change to be able to collaborate; external motivators and pressures towards collaboration such as supply chain problems, pressure from trading partners and availabilty of expertise; adaptability; development of appropriate policies and guidelines within the organisation
**Contextual/environmental** History of collaboration, local context, meeting the demands of the new environment
**Factors related to purpose** Objective reachable goals, common vision, specific and well-defined purpose, membership characteristics, common and agreed procees and outcomes
**Implementation climate** Political and social climate
**Cultural** Differences/similarities in goals and objectives, relationships, capacity to share risks, integration of key processes, flexibility of organisational system, compatibility of organisational culture
**Resources/physical** Investments, financial resources, funds, staff, expertise, skilled leadership

The themes identified from the theoretical framework were used to support the method of this study.

## Method

This study was conducted as part of an academic dissertation “Attitudes, perceived impacts and motivational factors for European Member State Collaboration for pricing and reimbursement of medicines: a review of the evidence,” submitted in partial fulfilment of the requirements of the Degree of MA in Management at the University of Malta. Raw data and further details of the methodology can be accessed from https://www.um.edu.mt/library/oar/handle/123456789/49528.

### Choice of Approach

**FIGURE 1:**
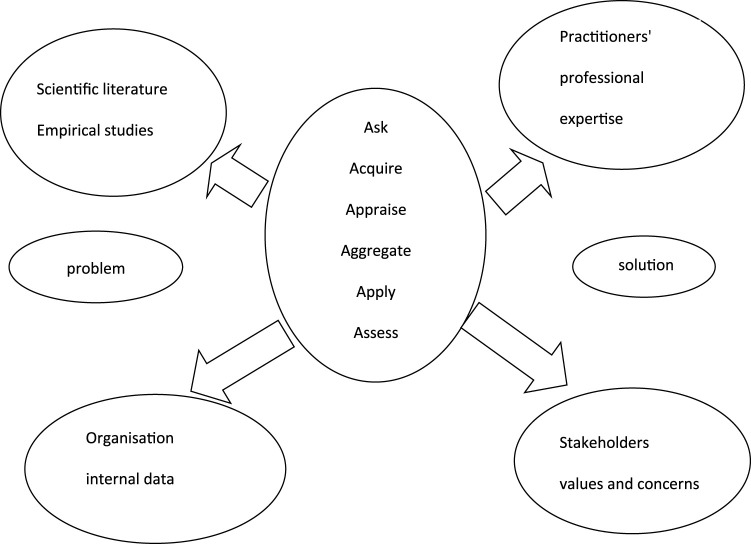
Model for the sources of evidence and for the steps for collection of evidence for evidence-based management decisions adopted from Barends and Rousseau (2018).

The study required a tradition of naturalism, whereby the researcher tried to understand the environment of the phenomenon under study and describe the setting and the interactions involved as they are. A design and framework specific to management interventions in the real life setting was chosen for this study. The approach for evidence-based management was used to gather information for this study. The sources of evidence as defined by Barends and Rousseau (2018) were used for the design of this study (refer to Figure 1).

Evidence-based management involves acquiring evidence about the specific subject under investigation from different sources including scientific literature and empirical studies, practitioners’ professional expertise and stakeholders’ values and concerns. The collection of evidence and its presentation involved six steps: ask, acquire, appraise, aggregate, apply and assess (Table 3).

**TABLE 3:** The process of this research.

Step of the process	Description
Step 1 Ask	• Description of the situation and challenges with Member State collaboration (the problem)
	• Defining the scope of the study
	• Presentation of the theoretical framework and relevant literature
	• Setting the research questions and the objectives of the study
Step 2 Acquire	• Setting the process for the study (presented in this Table)
	• Building a logic model of the process for pricing and reimbursement
	• Building a “Framework” for collection of the evidence for the concepts being studied: attitudes, perceived impacts (benefits and risks) and motivational factors (barriers and facilitators)
	• Using different methods to collect and present the evidence: scientific literature, grey literature, evaluation of “the Study,” focus group discussion
Step 3 Appraise	• Appraising of the evidence - critical appraisal of the evidence from each method for its trustworthiness and relevance
	• Evaluation of the balance and coverage of the evidence
Step 4 Aggregate	• Aggregation and presentation of the evidence from the different sources within the themes of the “Framework”
	• Corroboration and evaluation of the evidence from the different sources to see if there were gaps or paradoxes in the evidence
Step 5 Apply	• Inferences for the use of evidence-based management in practice
	• Application of the evidence for collaboration between Member State authorities for pricing and reimbursement
Step 6 Assess	• Use of the evidence to assess ongoing initiatives for Member State collaboration for pricing and reimbursement
	• Implications of the evidence for future initiatives for MS collaboration for pricing and reimbursement

### Methods for Acquiring Evidence

#### Choice of Methods

Initially three methods were identified to obtain the evidence: information from published scientific literature, analysis of grey literature and analysis of “the Study.” The mapping of the evidence which would be obtained from these methods showed that there was a lack of evidence which provided insight of the perspectives of practitioners from different MSs. Consequently, it was necessary to obtain primary data, and a focus group discussion with practitioners involved in MS collaborations was undertaken. The final mapping of the evidence obtained from the different methods is presented in Table 4.

**TABLE 4:** Mapping of the evidence collected through different methods with the four sources of evidence.

			Sources of evidence			
Methods			Scientific literature and studies	Organisation internal data	Practitioner professional expertise	Stakeholder values and concerns
1) Analysis of published scientific literature			X	—	X	—
2) Analysis of grey literature		Documents and reports	X	X	X	—
		Websites and documents of collaborations	—	X	—	—
		Conference proceedings	—	X	X	X
		WHO study on cross-country collaborations (WHO, 2020)	—	X	X	—
		Reports from stakeholders	—	—	—	X
		Media reports	—	X	—	X
3) Analysis of ‘The Study’			—	—	—	X
4) Focus group with practitioners			—	X	X	—

#### Collection of Evidence From Published Scientific Literature

This method was guided, as much as possible, by the methodology for systematic reviews. This evaluation of the scientific literature was performed by the first author (PVB). Search terms were identified using the PICOC and from the literature described in the *Introduction*. The search terms used were presented in Appendix 1.

The search terms were run through different databases (MEDLINE COMPLETE (EBSCO); Pro Quest ABI/INFORM Global; Cochrane database of systematic reviews; EBSCO host; SCOPUS; PLOS ONE; Psychology and Behavioural Science Collection). A number of search strategies were conducted. The PRISMA methodology for reporting of reviews (Moher et al., 2009) was used as a guide to present the literature review. The Critical Appraisal Skills Programme (CASP) checklist for qualitative research was used to evaluate the quality of the papers (Critical Appraisal Skills Programme 2018). The grey literature was also searched separately.

Articles published from 1st January 2000 to end May 2019 were considered. The inclusion criteria included articles directly relevant to the subject area (or related terms); articles related to the different activities for pricing and reimbursement and articles making recommendations for MS (inter) collaboration in their recommendations. Exclusion criteria included articles which covered cooperative partnership and networking between different stakeholders within the supply chain; articles focusing on techniques for evaluation, methodology, and procedures for pharmaceutical activities; papers describing cases for a specific medicine or medicinal product; activities such as manufacturing and the supply chain; articles which covered technologies other than human medicines; papers covering activities outside the scope of this dissertation such as pharmacy practice, prescribing, guidelines, supply chain, integrated healthcare networks, community care; and articles before 1st January 2000.

The number of articles for each search strategy was identified. The articles were screened through title and abstract. Those abstracts which possibly fitted the criteria and were directly relevant to the subject of collaboration and the abstracts which were possibly relevant for the building of the general logic model and for the description of the problem in the introduction were identified. Further screening was undertaken of the abstracts and further stratification was performed and duplicates were removed. Table 5 documents the number of papers retrieved.

**TABLE 5:** Summary of numbers of papers identified through the published scientific literature.

Total number of records: 85
No. of abstracts directly relevant to collaboration identified through database searching (duplicates removed) 78
Additional records identified through other sources: 7
- References as part of reports
- Articles identified during search for full-text articles
Number of full text articles found: 84
Abstracts without full text article: 1 (abstract in English, article in German)
Articles considered relevant during synthesis and included in the evaluation
45 full text articles and one abstract
Full text articles excluded: 39

Generally the articles up to 2014 gave more of a historical background and considered international collaboration as a wishful recommendation, with the exception of the EUnetHTA exercise which was considered primarily for the generation of guidelines and tools to be used by the MSs.

#### Analysis of Grey Literature

Organisational internal data to supplement the formal literature review was mainly obtained through hand searches, searching relevant websites and from reports of the MS entities for pricing and reimbursement activities and of the different regional collaborations. The cut-off date for collection of documents was the end of May 2019.

#### Analysis of the “Study on Impact Analysis for Policy Options for Strengthened EU Cooperation on Health Technology Assessment Final Report,” “the Study”

The main researcher (PVB) performed an analysis of the report of the stakeholder consultation conducted by the European Commission as part of the Better Regulation Exercise in preparation for the Proposal for a Regulation for Health Technology Assessment using the ‘Framework’ for this study. The evidence presented in the study was collected from different stakeholders, the specific stakeholder who submitted the information was also specified. While the scope of “the Study” covered pharmaceuticals, medical technologies and other technologies; for this study only pharmaceuticals were considered.

#### Focus Group With Practitioners in the Field

A focus group discussion was conducted to supplement the other methods. A professional European organisation was approached. The participants consisted of representatives from authorities and entities involved in pricing and reimbursement activities from different Member States. The President and the Secretary of the organisation accepted to include this focus group discussion as part of the agenda of a scheduled meeting which took place in Brussels in April 2019. There were thirteen participants for the focus group discussion. It was agreed that the researcher (PVB) was to record the discussion, prepare a transcript, circulate it to participants, obtain any feedback and their consent for future activities. The participants had diverse opinions on a number of aspects, and there were topics where there were clear opposing attitudes and critical views of certain activities. It was agreed that the transcript of the focus group discussion would not to be published. However, general inferences and observations could be made and included in future publications.

### Presentation of the Evidence From the Different Methods

The evidence for each method used was collated and analysed by thematic analysis (Bryman and Bell, 2011). Care was taken to include the context and the stakeholder perspective involved so as to limit any bias. For certain themes, specific indicators were included to give more detail and specificity. Information on the concepts of attitudes, perceptions on impacts and motivational factors for MS collaboration was used to build a “Framework” to support the collection and review of the evidence. This Framework consisted of a Logic Model of the process for pricing and reimbursement and tools with themes for the three concepts covered in the Research Question: attitudes, perceived impacts and motivational factors for MS collaboration for pricing and reimbursement. The theoretical framework on organisational theories, the knowledge on models for international collaboration, and the information for evaluation of attitudes, perceptions of impacts and motivational factors supported the drawing up of the “Framework,” which was used for acquiring, appraisal and aggregation of the evidence for the study.

The evidence from each of the methods was thematically placed in the “Framework” according to the themes. The classification of concepts was subjective and care was taken to keep it as standardised as possible. At times, the same concept could be fitted under different alternative themes depending on the perspective, the specific aspect and point of view considered e.g., IT infrastructure can be a challenge and can be a benefit, depending of the perspective. The themes used in the “Framework” and the indicators for the themes, supported the stratification of the evidence. The “Framework” was found to be comprehensive to represent the evidence collected from the different methods.

### Aggregation of the Evidence

As detailed in Table 3, Step 4 in the process for evidence-based management is “the aggregation of the evidence” to represent a final consolidated picture. Aggregation involved the weighing and pulling together of the evidence (Barends and Rousseau, 2018). The process sought to cover the different sources of evidence as comprehensively as possible. The level of corroboration of the evidence was evaluated and some gaps in evidence were identified. The level of robustness of the evidence was considered to give weight in prioritisation and in the determination of the impact of the different evidence. Perspectives by different stakeholders were noted and power mapping of stakeholders was performed. The aggregation of the evidence was presented as a structured narrative. The “Framework” with its themes and indicators was used to give a structure to the aggregation and to ensure the study questions were addressed.

The principal researcher (PVB) prioritised the information which was directly related to MS collaboration between pricing and reimbursement authorities. Care was taken to minimise subjective interpretation and selection of evidence. As this aggregation was based on the raw data collected, there was a second round of classification of the evidence under the relevant themes by the principal researcher (PVB), and some repositioning of the evidence was made. The method from which the evidence was obtained was noted to support cross referencing of the relevant information sources. Consequently, these were broken down as follows: SL – scientific literature; GL – grey literature; SIA – Study on Impact Analysis; FG – focus group.

### Application of the Evidence

In the discussion, the authors present their perspective of application of evidence for decision making, and present some recommendations for ongoing pharmaceutical strategy initiatives and challenges.

## Results

### Evidence Acquired From the Different Sources of Evidence

#### Scientific Literature

The papers identified from this review to cover specific activities of pricing and reimbursement are presented in Table 6.

**TABLE 6:** Activities of pricing and reimbursement covered in the published literature.

Activity for pricing and reimbursement	Papers specifically covering this activity
generation of evidence and sharing of information for HTA	Quentin et al. (2009); Rajan et al. (2011)
orphan diseases	Denis et al. (2010); Mincarone et al. (2017)
disinvestment	Henshall et al. (2012)
external reference pricing	Leopold et al. (2012); Vogler et al. (2017)
joint procurement	Huff-Rousselle (2012)
the experiences of WHO countries in joint procurement	Ferrario et al. (2017)
the evidence-based approach to decision making	Panteli et al. (2015)
horizon scanning	Douw and Vondeling (2006); Wild and Langer (2008); Nachtnebel et al. (2016); Oortwijn et al. (2018)
managed entry agreements	Bouvy et al. (2018)
real-world data	Garrison et al. (2007); Chatzidionysiou et al. (2018); Eichler et al. (2018); Geldof et al. (2019); McAuslane et al. (2019)
use of data to create a “learning healthcare system”	Eichler et al. (2018)
prices for orphan medicines	Luzzatto et al. (2018)

There were different types and quality of papers. No systematic reviews were identified in the literature review. In some published papers, collaboration was the main, or one of the main, objectives of the paper. Some papers were specific to the measurement of opinions about collaboration, used clear and specific methodologies for measurement of opinions, and reported the results through structured titles/themes.

Different methodologies were used though for the purpose of obtaining opinions. Some papers gave results from questionnaires or other methods within the paper either as the raw data or else in a collated manner. The presentation of raw data within a paper enabled direct evaluation of the results by the reader rather than just having access to the reporting and the interpretations given by the authors of papers. The interpretation between the authors of the paper and the reader could be different. In other papers, the opinion or recommendation for collaboration was made in an indirect manner.

Five papers clearly explained the methodology which they used usually involving both quantitative and qualitative information and the results were presented in a comprehensive manner. These papers used questionnaires for project participants and for external stakeholders to evaluate the experience of EUnetHTA first Joint Action and gave the results in a global manner. Kleijnen et al. (2015) gave detailed results from semi-structured interviews for the evaluation of opinions on collaboration for relative effectiveness assessment (REA) with representatives from eight HTA organisations (Kleijnen et al., 2015). The results from this paper were very congruent with the responses of the MS Representatives in the “The Study.” Rajan et al. (2011) evaluated motives, enablers and barriers to the promotion of HTA mainly through a two-phase study using a questionnaire and compared responses on enablers and the prioritisation of enablers across context and cultures (Rajan et al., 2011). Henshall et al. (2012) summarised the main points from presentations, discussions among attendees at conferences and produced an advanced background paper at an international meeting (Henshall et al., 2012). Panteli et al. (2015) extracted information from online sources (Panteli et al. (2015).

Three papers were “perspectives” papers: Ferrario et al. (2017), Vogler et al. (2017) and Vella Bonanno et al. (2019). These papers did not adopt a standard method but involved a presentation of ideas and alternative opinions. It was difficult to classify such papers using the CASP tool. On the other hand, these papers were enlightening in terms of giving insight and bringing out perspectives which went beyond just facts and direct questions. Consequently for the purpose of this study, these three papers were considered as useful for the generation of evidence. Two of these perspectives papers were written jointly with staff from WHO and showed perspectives about contemporary issues which were impacting outcomes related to medicines: Vogler et al. (2017) dealt with policies for pricing and reimbursement and Ferrario et al. (2017) gave perspectives on strategic procurement (Ferrario et al., 2017). Vella Bonanno et al. (2019) gave perspectives on the “Proposal for a regulation on health technology assessment” from 36 policy makers, payers and academics from the field of HTA (Vella Bonanno, 2019).

Eight papers reported actual experiences of collaboration and gave a description of personal or third-party experiences of practitioners who worked in organisations. Some papers went to the level of reporting the achievement or progress of the collaboration. These papers were of high quality in relation to the CASP tool and had clear objectives related to the collaboration through existing projects, mainly the EUnetHTA project. These papers were presented in a structured way with clear methodology and five of these papers were published in the same journal. The greatest experience in collaboration to date was regarding activities of the EUnetHTA JA projects. A number of papers were published between 2010 and 2014 coinciding with the first EUnetHTA Joint Action (JA 1), which dealt with the development of tools and the EUnetHTA Core Model. These included Quentin et al. (2009); Lo Scalzo et al. (2014); and Woodford Guegan and Cook, (2014). The highest volume of papers with experience on collaboration concerned REA and these papers were mainly published between 2014 and 2016 at the same time as the second EUnetHTA JA. These included Huic et al. (2013); Woodford Guegan et al. (2014); Panteli et al. (2015); and Mayer et al. (2017). The paper by Nachtnebel et al. (2015) gave a deeper insight and brought out points about the challenges with the adoption of HTA assessments at the national level (Nachtnebel et al., 2015). The paper by Erdos et al. (2019) gave a more recent update and was highly insightful (Erdos et al., 2019).

A number of papers focused on specific activities related to pricing and reimbursement. These papers often made no specific recommendations for collaboration as a means for the improvement of the activity. In these papers, it was difficult to apply the criteria of the CASP tool directly. The objectives of these papers were the activities and not collaboration. There was a pattern of activities over time. There were activities with good build up of experience (e.g., REA), while some activities such as real-world evidence gathering and disinvestment were still being developed, defined and described.

Four papers described initiatives for national HTA in different countries, mainly at the time when the specific country was undertaking initiatives for improvement/development of their national system e.g., Belgium: Cleemput and Van Wilder (2009); Hungary: Kalo et al. (2013); Greece: Souliotis et al. (2016) and Slovakia: Tesar et al. (2017). These papers were mainly descriptive and made recommendations for collaboration in the future.

Four papers compared differences and similarities in considerations and recommendations by HTA agencies in different countries during HTA evaluations. These included Kleijnen et al. (2012); Oyebode et al. (2015); Panteli et al. (2015); and Allen et al. (2017). Makady et al. (2017) made a case for real world collaboration for real world data (Makady et al., 2017). One paper described the benefit of the use of the HTA Core Model by a pharmaceutical company (Ducournau et al., 2019).

Some papers included in this review were used to update the Logic Model, for example: horizon scanning systems in Douw and Vondeling (2006) and Wild and Langer (2008); reference pricing in Leopold et al. (2012); horizon scanning in Packer et al. (2015), and experience of HTA agencies in Loblova, (2016). Few papers specified outcomes, for example Zaprutko et al. (2017) measured affordability.

Some authors were involved as main authors and/or co-authors in more than one paper. This led to a level of standardisation in the presentation of the papers e.g., the papers on the outcome of EUnetHTA Joint Actions.

Reference price systems were considered as initiatives for MS collaboration because there were organised systems for exchange of information on prices and on policies.

The highest reason for exclusion of papers was in cases where papers dealt with or recommended collaboration between different stakeholders (interdisciplinary collaboration) e.g., payers, life-sciences companies, industry, and stakeholders within the supply chain; and did not cover international cooperation between MSs. One paper was excluded because it considered collaboration across healthcare services in councils within the same country. Another reason for exclusion of papers were papers which only considered countries outside the European Union. The EuroScan network was not considered as a MS collaboration for pricing and reimbursement because it consists of an international network of publicly funded agencies undertaking horizon scanning which are not necessarily national agencies for pricing and reimbursement but include different types of agencies such as public health agencies. Members of EuroScan were often service providers for Pricing and Reimbursement agencies (Packer et al., 2015). EuroScan was included in the Logic Model.

A number of papers considered technical aspects of pricing and reimbursement but did not consider collaboration between MSs. Examples of aspects considered included medicines adaptive pathways, pricing frameworks, aspects of decision making for HTA, e.g., multiple criteria decision analysis, sequence of activities for reimbursement decisions, personalised medicines, medicines availability and affordability and big data.

A number of papers covered comparison of the requirements, similarities and differences between reimbursement evaluations in different countries. These papers were included if they linked these aspects to recommendations regarding collaboration. There was also increased literature about experiences of disinvestment in different countries.

#### The Grey Literature

A summary of the different types of documents used as grey literature and the sources of evidence served is predented in Table 7.

**TABLE 7:** Different types of documents used as grey literature and the sources of evidence served.

Type of document	Scientific literature and studies	Organisation internal data	Practitioners‘ professional expertise	Stakeholder values and concerns
Policy Briefs	X	X	X	—
Council of the European Union e.g., Council Conclusions	—	X	—	—
Research study on impact and benefits of cross border collaboration in WHO European region, (WHO 2020)	—	X	X	—
International Conference “Facing the Challenges: Equity, sustainability and access” (INFARMED 2018)	—	X	—	—
Information from the Regional cross-country collaborations (websites, press-releases)	—	X	—	—
Interdisciplinary Platform on Benefit Assessment	—	—	—	X
Innovative Medicines Initiative	—	—	—	X
On-line magazines	—	X	—	X
Patient Associations	—	—	—	X
Industry opinion on cross-country collaborations	—	—	—	X

Overall, the sourced grey literature was very useful to study different perspectives and in particular in-depth insights from different stakeholders; although, in some cases the evidence could not be directly linked to a specific stakeholder. A number of documents from the grey literature represented the perspectives and priorities of organisations: individual member states or collective opinions. The media gave information and insights of happenings which would otherwise have remained hidden. Often journals used the tactic that they reported information from someone (e.g., an insider of the organisation) who was not allowed to divulge information.

The Policy Briefs typically gave a comprehensive and balanced overview of the relevant topic. Policy briefs mainly adopted the MS’ perspective/s. Council Conclusions gave joint positions from the MS. Conferences and conference proceedings were particularly relevant because they gave an overview of a topic and also provided an insight about evidence from sources of information, such as organisational information or stakeholder perspectives, which was typically not published in scientific literature unless the conference proceedings were published as a Supplement to a Journal. A few of the documents included in the grey literature were prepared and organised according to systematic methodology; these included studies contracted out through bodies such as WHO, for example the “Research study on impact and benefits of cross border collaboration in WHO Europe region” by World Health Organisation (2020) and the Policy Briefs.

From the evidence, it was difficult to clearly qualify and quantify the achievements and outcomes from the activities of the MS collaboration. In reality, there were few significant achievements beyond sharing of information.

#### “The Study”

The “Study on impact analysis for policy options for strengthened EU cooperation on HTA, Final report” (European Union 2017), referred to as “The Study,” was the most robust study identified which measured the perceptions on impact of MS collaboration. It contained feedback from different stakeholders. One main limitation of “The Study” was that it was limited to only one activity, i.e., HTA. “The Study” used robust methodology and the methods used to collect evidence were well documented. The main objective of “The Study” was to evaluate sustainable cooperation for HTA beyond 2020.

Detailed analysis of “The Study” revealed some limitations. The Commission pre-conditioned the options with its criteria and did not present a blank drawing sheet. The authors of “The Study” adopted the Policy Options and conditions set by the Commission. “The Study” did not consider an implementation mechanism without EC funding because the Commission considered that intergovernmental collaboration without input from the EU was strictly the responsibility of the Member States. The authors of “The Study” expressed their collective opinion about specific issues in quite a definitive and specific manner; and this could bias the output of “The Study.”

The presentation of the results was based on the Better Regulation framework of impacts and the impact analysis focused on impacts. Greater emphasis was placed on the economic impact rather than the social impact. This reflects the priorities and perspectives of stakeholders, with companies typically more concerned with economic rather than social impacts.

The response on impacts was presented according to different stakeholder groups; however, not all stakeholder groups responded. The pharmaceutical industry and public administrations provided most of the feedback. The point of view of these stakeholders (particularly the industry with most respondents) overpowered the results and also the recommendations. The recommendations of these two major stakeholders differed, with the recommendations of the industry dominant and most reflected in the final legislative proposal. Not all countries responded to this study and consequently the opinion of the MSs as reported in “The Study” did not reflect certain opposing views which were later expressed during the discussion of the Proposal for a Regulation on HTA at the European Council.

The participation in the survey by patient organisations was far too low and the authors decided that they could not analyse the feedback from the patient response. Consequently, nothing was included on the patient response in the summary of the results. However, the detailed report had some information on the perspectives of patient associations, where concern was expressed about uncertainty due to conditional approval. This response was very significant and very contrasting to the feedback by the industry.

#### Focus Group With Practitioners in the Field

The participants at the focus group considered the topic of collaboration as sensitive and political, particularly in view of the discussions on the Proposal on a Regulation on HTA which at that time was being discussed at Council. There was a general positive consideration on collaboration for certain activities for pricing and reimbursement including horizon scanning and collection of real-world data but not for HTA. There was a divergence of opinion on the experience and success of regional collaborations. Some participants came from countries which were involved in a regional collaboration while some participants were from countries that were sceptical of such collaborations. The discussion about the regional collaborations was quite challenging. There were in-depth insights regarding the politics and the challenges being faced in the interaction between the industry and the Member States within the regional co-operations, and this is likely to remain.

### Updated Logic Model for the System of Pricing and Reimbursement

**FIGURE 2:**
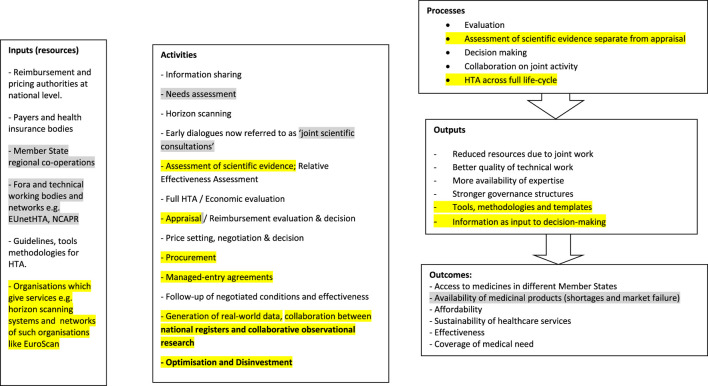
Final Logic Model for the system of pricing and reimbursement. Grey highlights: updates from the scientific literature, Yellow highlights: updates from the grey literature.

The evidence on the different processes for pricing and reimbursement from the different methods was used to update the Logic Model for the system of pricing and reimbursement. The final and updated Logic Model for the system of Pricing and Reimbursement as in September 2019 is presented in Figure 2.

The updates to the Model which were derived from scientific literature were highlighted in yellow while the updates derived from grey literature were highlighted in grey.

There were a number of changes in the process for pricing and reimbursement, including collaboration between pricing and reimbursement authorities, which was a relatively new concept. More recently there was a high level of initiative for “coordinated collaboration alongside the life-cycle” (Eichler et al., 2018; Vogler et al., 2018) and this required collaboration between different stakeholders both at national level as well as across countries.

### Aggregation of the Evidence

As discussed in the Method, during aggregation of the evidence, the evidence was referenced according to the source of the evidence: SL: scientific literature; GL: grey literature; SIA: Study on Impact Analysis, “The Study”; FG – focus group.

#### Attitudes on Collaboration Between National Health Authorities for Pricing and Reimbursement

A main theme concerning attitudes on collaboration was whether collaboration should be voluntary or mandatory. The policy options for the Proposal for a Regulation on HTA presented different options for the type of participation and for the uptake of joint outputs (SL; SIA). In 2016, The Council of the European Union recognised that a number of MSs expressed interest in pursuing voluntary cooperation and stressed that these activities should remain voluntary and be focused on added values (GL). The European Commission proposed that collaboration would be voluntary for all activities except for HTA (GL). During the focus group discussion, a number of practitioners were vociferously supportive of voluntary collaboration and against mandatory collaboration. The opinion towards mandatory collaboration was overshadowed (FG). “Strong” countries which have well established systems for pricing and reimbursement considered that they did not gain from collaboration; in fact they stood to lose (FG; GL).

The stakeholders had differing opinions on collaboration on HTA. The authors of “The Study” considered that economic impacts were particularly relevant to the industry; sustainability of healthcare systems was mainly relevant to public administration authorities and social impacts were relevant to citizens, patients and healthcare professionals (SIA). The industry considered that collaboration on HTA would be positive for mandatory participation and uptake of REA but would be negative for full HTA, i.e., the economic part of the assessment was not to be considered because of the high level of agreement that would be needed. Industry graded legislation for full HTA (including REA) negatively for all themes (SIA). In “The Study,” public administrations were reported to favour full HTA including the economic part with a legislative framework and mandatory participation and uptake (SIA). Patients also favoured policy options with mandatory participation and uptake as these were considered to increase the availability of medicines and to ensure standardised monitoring of health technologies prior to market access (SIA).

Collaboration could take place at different levels, ranging from loose collaboration such as exchange of information and development of common methodologies, to joint collaboration on cross-border assessments (SIA). Traditionally (up to 2015) the MSs collaborated by setting joint tools, guidelines and methodologies which were subsequently to be used at a national level, bringing a level of standardisation but allowing for differences between MSs. The experience of the project-based cooperation of EUnetHTA resulted in a number of challenges. These included the fact that joint work was used to a limited extent by the MSs, there was low uptake of joint work by national HTA authorities mainly due to legal and administrative hurdles, there were concerns on quality assurance, the joint timelines needed to be aligned with national timelines, and there was lack of sustainability of the work produced at the project setting (GL). More recently, it is preferred to undertake working together as one output (SL; SIA; GL). While practitioners seemed to generally agree on the technical aspects related to tools, guidelines and methodologies for HTA, at the political level there was divergence of positions on joint work, which were more at the level of national politics than the perspectives of individual practitioners (FG).

As mentioned, “The Study” showed that the European Commission and the authors of “The Study” considered that intergovernmental cooperation without input from the European Commission was not to receive EU funding and was strictly the responsibility of the MSs (SIA). Practitioners considered that the Proposal for a Regulation on HTA, as published, was much more supportive of the economic rather than the public health perspective. They felt that with the introduction of a Regulation for HTA, the position of the MSs within the power balance will decrease as compared to industry. Practitioners had mixed opinions on the power position. Those who felt less influential and powerful in the MS hierarchy (the “weaker” MSs) preferred to have legislation which brought all MSs at par rather than them being bullied by “stronger” MSs. MSs which had well-established HTA systems and had negotiating power (the “stronger” MSs) strongly resisted mandatory collaboration. Practitioners felt that there were advantages from collaboration including production of evaluations of high quality. They considered that the European Commission proposed collaboration which was “coercive.” Practitioners believed that power within HTA collaborations would be restricted. If the methodology for HTA including the criteria for REA were to be set through legislation, as set in the original Proposal, this was considered to restrict the collaborators from evolving and adapting the methods. MSs were concerned with transferring the authority for REA outside national jurisdiction and the Proposal was considered to take over national legislation (SL).

As stated earlier, during the focus group discussions, there were divergent opinions with respect to regional collaborations, particularly with regards to their outcomes and success. There was consensus though that the industry was not typically willing to participate in joint negotiations (FG). The regional collaborations were considered as the reaction of the MSs to the industry’s game of divide and rule (GL).

In 2019, the main innovative industry association (EFPIA) considered that some initiatives for collaboration could support increased access to medicines and sustainability of healthcare systems, while in some cases national procedures were preferred. EFPIA considered that collaboration was in its infancy and there was little successful experience of enhanced access to medicines through collaborative initiatives. EFPIA suggested that until there was evidence of benefit from collaboration, national access processes were to remain the preferred way for timely access (GL). In 2018 the media criticised the BENELUXA cooperation because of lack of concrete negotiation deals (GL). The BENELUXA stated that at times industry did not want it publicly known that negotiations were underway (GL).

MSs had a general positive attitude for voluntary collaboration for different activities, with the exception of a clear divergent position on voluntary/mandatory REA. The scientific literature was the main source of information to inform of or promote new activities such as the generation of real-world data. MSs tended to keep the information on their actions and initiatives for collaboration internal e.g. the building of the horizon scanning initiative within BENELUXA and the regional collaborations kept the information within their organisations. There was consensus from the different sources that sharing of information on HTA was the first step towards collaboration, and the one which is most likely to be successful. The attitudes on other activities are not so clearly stated, probably because there was uncertainty on the possible level of achievement of outcomes. The MSs participating in regional collaborations were more interested in activities which they considered to increase access to medicines, particularly joint negotiation. The industry was supportive of mandatory REA and early dialogues but not of the other activities. Patients were mainly focused on the attitude that there should be patient involvement in activities and in decision making.

#### Perceived Impacts (Benefits and Risks) From Collaboration Between National Health Authorities for Pricing and Reimbursement

The themes of the “Framework” and the indicators for each theme for the perceived impacts supported the strategic aggregation of the evidence from the different sources and standardisation with the relevant theme. The themes were divided into social health impacts and economic impacts in line with the Guidelines for Better Regulation of the European Commission. At times, the same information could fit into different themes.

The scientific literature mainly reported the positive experiences and perspectives (benefits) in relation to improved quality of output and the benefits from joint methodology and tools. “The Study” specifically studied the perspectives of the impact for different stakeholder groups and was the most informative method in this respect. “The Study” showed that there were conflicting perspectives between and within stakeholder groups. The industry (and also the authors of “The Study”) considered economical benefits as key, while the MSs emphasised the social impacts such as access, sustainability of health care systems and public health. “The Study” considered that there would be improved governance, improved quality of assessment, standardisation of tools and methodology and reduced administrative burden for MSs (SIA). Contrastingly the scientific literature (Vella Bonanno et al., 2019), the focus group discussion, and the grey literature, showed that while the countries with no resources favoured collaboration, countries which had well-established systems for HTA considered collaboration as a deterrent. Countries with resources feared that they would lose their autonomy and potential for local contextualisation and possibly it would take longer to come to a concerted assessment, leading to waste of time for access to medicines. The latter perception was also being reflected by a number of MSs during the actual discussion of the Proposal on HTA at Council. It was important to note that the perspective of the “stronger” MSs was not expressed in “The Study.” Industry classified all themes for joint economic assessment as negative while the MS representatives were supportive of joint economic assessment. The final Proposal for a Regulation on HTA did not include joint economic assessment.

#### Negative Motivational Factors (Challenges/Barriers) and Positive Motivational Factors (Drivers/Facilitators) for Collaboration Between National Health Authorities for Pricing and Reimbursement

The themes in the “Framework” were considered adequate for the presentation and aggregation of the available evidence on motivational factors. There was some overlap between the concept of motivational factors and perceived benefits and risks. The grey literature was the main source of evidence on motivational factors and gave an insight mainly from the point of view of MS organisations. There seemed to be more evidence on challenges than facilitators for collaboration. The main driver for collaboration for MSs was increased access to medicines. The main challenges were not of a technical nature but concerned mainly cultural, national and political factors such as safeguarding national jurisdiction, autonomy over activities for pricing and reimbursement, problems with harmonisation across MSs, the national level of engagement, building of trust, national legislations, the need for specific resources for collaboration and political will and commitment.

## Discussion

### Application of the Evidence

The aggregation of the evidence from the different methods and its the corroboration enabled the building of a holistic and realistic picture.

There was an imbalance in the publication of evidence. A great deal has been published about the Proposal for a Regulation on HTA; however, less has been published regarding certain new activities related to pricing and reimbursement. The generation of evidence came in waves, for example the concept of real-world data in response to conditional marketing authorisation was still evolving and the evidence followed the same patterns. There was also the limitation that confidential data, such as minutes of meetings, which could not be used.

The aggregation of the evidence from different sources showed that some individuals and/or organisations participated in more than one initiative, and there were “opinion leaders.” Certain activities such as funded projects and high-level meetings between different stakeholders could be used to change the political balance in relationships and possibly reorganised the power between stakeholders.

Although grey literature is generally considered as lower quality of evidence in terms of the hierarchy of evidence for HTA, for the purpose of evidence-based management it proved to cover a more holistic picture and was particularly useful to show organisational insights and professional opinions. It is recommended that a different “pyramid for the hierarchy of evidence” is drawn, specific for the study of attitudes and of perspectives, with methods that provide insight being ranked high in this pyramid. The media was specifically useful to uncover the controversial points and to bring them to light. The media was like a balance check for the evidence, although it was still biased. The focus group discussion was probably “too good” a method to bring out the issues, so much so that the participants decided to block it. This gave an indication of what may actually happen in reality, and with the exception of the media (where there is still an element of lobby and alliances), the evidence which gives too much insight of reality may tend to get blocked.

‘The Study’, which followed the methodology of EU Better Regulation, focused on the perceived impacts of the intervention. It did not consider the attitudes of the main stakeholders and the challenges and motivators related to the intervention. This is considered as a deficiency of the EU Better Regulation methodology of the European Commission.

One major lesson learnt from this research was the risk of bias when reading literature and interpreting the various studies. When “The Study” was published, the first author (PVB) had given it a good viewing. After a thorough evaluation of “The Study” as part of this research, it was clear that although the methodology of “The Study” was clear and according to the rules, the final outcome of “The Study” gave a strong prominence to the position of industry and made less emphasis to certain points made by other stakeholders such as the patient organisations, which had much less representation.

Generation and processing of the evidence was very time consuming. It would be difficult to generate such a lot of evidence in the future to address decision making in routine practice. This methodology would probably be limited to very important decisions, as were the decisions on initiatives for Member State collaboration for pricing and reimbursement.

### Assessment of the Evidence for Ongoing Pharmaceutical Strategy Initiatives

The main aspects of attitude for collaboration were voluntary/mandatory participation in collaboration, the uptake of the joint output from collaboration, and the attitude towards the formation of regional collaborations and different perspectives of stakeholders.

This research showed that the power and attitude of the MSs involved will determine whether countries will be willing to collaborate together and which model of organisational structure will be chosen for the collaboration The differences in attitude on voluntary/mandatory collaboration between the “stronger” MSs and the “weaker” MSs could be explained in terms of motivational factors. The “stronger” MSs considered that their economic strength gave them enough power for negotiation with the industry, and thus they were not motivated to collaborate with other MSs, while “weaker” MSs needed to build power for negotiation through grouping. This is aligned with Baldwin et al. (2012) who stressed that collaboration requires the building of organisational structure (Baldwin et al. (2012). From the evidence on attitudes gathered it was clear that all MSs had a negative attitude for “hierarchical” modes of collaboration. The regional co-operations showed that a number of countries were willing to collaborate as a “network” as described by Baldwin et al. (2012). Networking is recommendable, as long as there are concerted efforts for agreed actions.

The power and attitude of the MSs involved will determine whether countries will be willing to collaborate together and which model of organisational structure will be chosen. Forming smaller groups, such as the regional cooperations, may make it easier for the collaborating MSs to find an aligned scope and benefit. The regional co-operations are being formed by MSs voluntarily and therefore these only form if the MSs feel that they are mutually benefitting from the collaboration in one way or another. It is clear from the evidence that inititiatives for collaboration must be introduced voluntarily and that MSs are to be left to participate out of their own will.

A high emphasis was placed by certain stakeholders particularly the European Commission and the industry on benefits for “governance and administration” in terms of improved quality of assessment, with standardisation and harmonisation. This is in line with the results of the systematic review by De Freitas et al. (2018) which ranked governance, participation and good administration as high impacts. Other sources of evidence, particularly the grey literature and the focus group discussion, showed that from the perspective of the MSs the main benefits from collaboration were improved public health outcomes, particularly increased access to medicines, rather than improved outputs, i.e., governance and good administration. While the healthcare systems of the MSs differ, and there are different levels of affordability, all MSs have challenges with access to new medicines (to different extents) and all support the concept of collaboration to increase access to medicines.

The results of this study showed that “factors related to purpose,” cultural factors, and the implementation climate were key motivators for MS collaboration. The factors identified in the results of this study were aligned with the factors mentioned by Kezar (2006) who identified culture, shared values, relationships and priority from senior management as main motivational factors for collaboration between educational institutions (Kezar, 2006). Different stakeholders had different purpose in relation to MS collaboration. The results showed that there is a major difference in the motivation and the purpose of different stakeholder groups. The most distinct differences in pupose was seen between the MSs and the industry e.g. in relation to transparency of prices. Patient organisations may also get involved in this challenging area. This impacts the level of trust between stakeholders, the perceptions of any initiatives for collaboration and the level of trust in authorities who take initiatives to bring about collaboration e.g., through the setting of new policies and strategies.

Kezar (2006) recommended that successful implementation of collaboration involves redesign and learning of collaboration skills and unlearning of non-collaborative practices (Kezar 2006). This may be a reason why the practitioners in the larger and well-established organisations were reluctant to collaborate; they were resistant to redesign and change what they had, over years, painstakingly built to minimise risks. Moreover, well established systems support practitioners to build their niches and experts may not want to lose their prima-donna positions within their organisations and to get diluted within a pool of experts.

As shown by Koeing-Archibugi (2010) one reason for international cooperation may be a blame-management and blame-shifting incentive and ministers may want/need to show that they are tackling the challenge of access to medicines (Koeing-Archibugi 2010). This may be part of the motivation for all MS governments to participate in European-wide procurement of COVID vaccines.

The principle of bounded rationality (Simon 1991) considers that one individual or organisation has limited capacity to process all information within the existing constraints; resulting in decisions being inherently bounded. These benefits of collaboration are also exhibited by the regional collaborations, whereby collaboration helps to overcome the limitations of individual MSs. and shows potential to improve decision making.

One bold consideration for the regional collaborations is for the MS involved to introduce transparency of prices of medicines between themselves. It is not possible for all the Member States to be gaining from the current confidentiality on prices. The Member States know the published (list) prices of each other, which in reality may not be the true price when considering different forms of discounts involved. Transparency of information on prices should be between the countries that agree to the terms of the collaboration and should not extend beyond the countries participating within the regional collaboration because of the system of reference pricing. It may also be problematic for patients if co-payments are based on list rather than actual prices. In addition, concerns whether confidential discounts will be seen as undemocratic as debates within parliaments are not possible when ministers have already negotiated confidential discounts (Godman et al., 2021).

In spite of progress achieved, MSs still have much to learn about the possible benefits and synergies from collaborating together. Strong political will is needed as there can be a lack of trust and of motivation among MSs to collaborate. Consequently, the ongoing collaborations need to take bold steps to surmount the main hurdles and fears. They need to be assertive, they need to trust each other and they must address fears of the unknown. It takes time to build trust and political will. Ideally the main ongoing regional collaborations will take actions at the same time, if not jointly. The positive experience and the willingness to collaborate exhibited by MS during the current COVID pandemic shows that where there is strong motivation hurdles can be overcome.

## Conclusions

The method of evidence generation described by Barends and Rousseau (2018) is applicable to generate evidence which can be used for decision making at the management and policy level.

The evidence from this research can be used to inform future decisions about initiatives for MS collaboration. The project shows that different MSs have different levels of motivation to collaborate on different activities and the main determining motivational factor for realisation of collaboration is political. This information corroborates well with the level of difficulty encountered during the discussions on the Proposal on HTA and the way that joint procurement for the COVID pandemic was handled.

Member States already have extensive experience and knowledge in applying evidence for decision making with respect to pricing and reimbursement of medicinal products within their national health care systems. They should apply the principles of evidence-based practice to their management and to policy decisions too. The Logic Model for the system for pricing and reimbursement (Figure 2) shows the various activities in the area of pricing and reimbursement of medicines and the considerations for collaboration differ for the different areas. There is already established cooperation in areas like horizon scanning and use of real world data. Collaboration for HTA evaluation will eventually be covered by the anticipated new Regulation on HTA, which should reflect the agreed MS position. There are other initiatives which can be considered such as collaboration to increase transparency of prices between MS and collaboration on procurement, particularly following the experience of joint procurement during the COVID pandemic.

Stakeholders within the pharmaceutical framework have contrasting interest and whenever a strategic decision is to be taken, understanding the different political positions and powers of the stakeholders concerned is key and influence/interest mapping should be undertaken formally using the best evidence and incorporating the involvement of the different stakeholders concerned. This will be the basic principle for MS to consider when they need to establish collaborations with other stakeholders such as the pharmaceutical industry.

Currently, there are a number of ongoing initiatives for setting of a pharmaceutical strategy by the different authorities including the European Commission (Pharmaceutical Strategy for Europe, 2020 and European Commission proposals for “A European Health Union: Tackling health crises together”) and the World Health Organisation. Now is the time to look at the legislative and policy aspects which could have hindered initiatives for collaboration in the past and to address them. The multitude of ongoing concurrent initiatives may lead to overlap in policy or conflicting directions. Ideally there is collaboration at the policy and strategy level as well.

## Declarations

Patricia Vella Bonanno works with the Ministry for Health in Malta and is a member of the Valletta Technical Committee. All opinions expressed in this publication express her personal positions as an academic and are not representative of her post.

Patricia Vella Bonanno and Brian Godman are members of the Piperska Group and have participated in joint publications as part of this Group. The opinions expressed in this publication are personal and not in the name of the Piperska Group.

This study was conducted by Patricia Vella Bonanno for a dissertation “Attitudes, perceived impacts and motivational factors for European Member State Collaboration for pricing and reimbursement of medicines: a review of the evidence,” submitted in partial fulfilment of the requirements of the Degree of MA in Management at the University of Malta. Vincent Cassar was the main academic tutor for this dissertation. Brian Godman contributed as pharmaceutical expert for the dissertation as well as for this publication. The full dissertation including the raw data (presented in its Appendices) is published by the Library of the University of Malta on https://www.um.edu.mt/library/oar/handle/123456789/49528.

Ethical Clearance for the study was carried out as part of the requirements of the Dissertation on the 29th of May 2019 by the Faculty Research and Ethics Committee of the Faculty of Economics, Management and Accountancy at the University of Malta.

## Data Availability

This study was conducted as a dissertation by PVB. The full dissertation, including the raw data is published by the Library of the University of Malta and is accessible on https://www.um.edu.mt/library/oar/handle/123456789/49528.

## Appendix 1

**Table:**

Search Terms Used for the Collection of Evidence from Published Scientific Literature	
**Area**	**Search terms**
Collaboration	Collaboration, cooperation, inter*, network; cross border; European
Activities for pricing and reimbursement	Early dialogue; scientific advice; Health technology assessment; pric*; reimbursement; horizon scan*; procurement; real world data; pharm*; medicine
Attitudes	Attitude; belie*; perspective; critical success factors
Impacts	- benefit
	- risk, loss
Motivational factors Barriers and facilitators	- influencers
	- barrier, challenge, obstacle
	- facilitat*, motivat*, driver, bridge
